# Radial nerve palsy in the newborn combined with congenital radial head dislocation: Case report and literature review

**DOI:** 10.1097/MD.0000000000037146

**Published:** 2023-02-02

**Authors:** Yunlong Li, Guoxin Nan, Jiahui Chen, Yongyao Jiang, Weiyue Zhu

**Affiliations:** a Pediatric Orthopedics, Dongguan Children’s Hospital, Dongguan City, China; b Intensive Care Unit, Dongguan Nancheng Hospital, Dongguan City, China.

**Keywords:** brachial plexus palsy, congenital, newborn, radial head dislocation, radial nerve palsy

## Abstract

**Rationale:**

Radial nerve palsy in the newborn and congenital radial head dislocation (CRHD) are both rare disorders, and early diagnosis is challenging. We reported a case of an infant with concurrent presence of these 2 diseases and provide a comprehensive review of the relevant literature. The purpose of the study is to share diagnostic and treatment experiences and provide potentially valuable insights.

**Patient concerns:**

A newborn has both radial nerve palsy and CRHD, characterized by limited wrist and fingers extension but normal flexion, normal shoulder and elbow movement on the affected side, characteristic skin lesions around the elbow, and an “audible click” at the radial head. The patient achieved significant improvement solely through physical therapy and observation.

**Diagnoses:**

The patient was diagnosed with radial nerve palsy in the newborn combined with CRHD.

**Interventions:**

The patient received regular physical therapy including joint function training, low-frequency pulse electrical therapy, acupuncture, paraffin treatment, as well as overnight splint immobilization.

**Outcomes:**

The child could actively extend the wrist to a neutral position and extend all fingers.

**Lessons:**

If a neonate exhibits limited extension in the wrist and fingers, but normal flexion, along with normal shoulder and elbow movement, and is accompanied by skin lesions around the elbow, there should be a high suspicion of radial nerve palsy in the newborn.

## 1. Introduction

Isolated peripheral neuropathy of the upper extremity in the newborn is rare compared to brachial plexus palsy, with fewer than 80 cases of isolated radial nerve palsy in newborns reported worldwide. The condition may often be misdiagnosed as brachial plexus palsy due to lack of awareness, but usually does not have adverse outcomes due to its favorable prognosis. CRHD is also rare, but it is the most common congenital deformity of the elbow. Unlike neonatal radial nerve palsy, CRHD is uncommon in newborns and often remains asymptomatic until puberty, when it is not diagnosed until limited elbow movement or pain occurs. When these 2 rare diseases occur simultaneously in a child, it seems to be more unique, and we have reported on it and introduced the treatment and prognosis.

## 2. Case presentation

**Figure 1.**
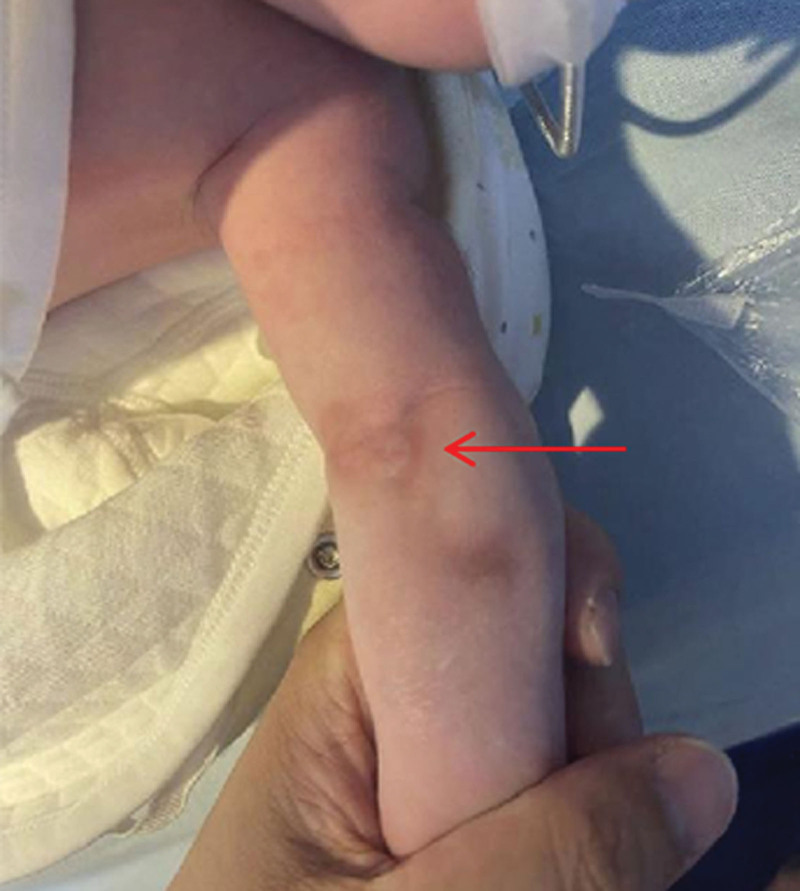
A 1.5*2 cm bruise was observed on the anterolateral aspect of the left elbow (red arrow).

The publication of this case report obtained written informed consent from the patient legal guardian. A 38-year-old mother, G2P1, delivered a male baby via cesarean section at 40 + 1 weeks of gestation, weighing 3630g. Physical examination revealed that the baby had left forearm pronation, left wrist and fingers hanging down, no active extension movement, but a grasp reflex could be in the left hand. The left elbow and shoulder joints showed autonomous movements. A 1.5*2cm bruise and palpable lump were observed on the anterolateral side of the left elbow (Fig. 1). After ruling out infection, clavicle and upper limb fractures, a preliminary diagnosis of radial nerve injury was made. We immobilized the infant left wrist in a neutral position with a splint. Subsequently, the family chose to forgo further examinations and voluntarily discharged the baby.

**Figure 2.**
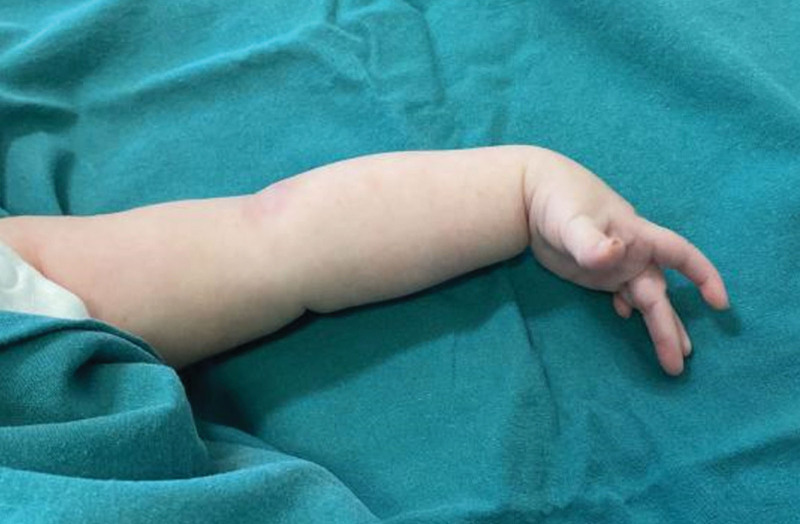
A momentary extension of the fingers was observed, with overall weak strength and inability to reach neutral position in the middle finger.

**Figure 3.**
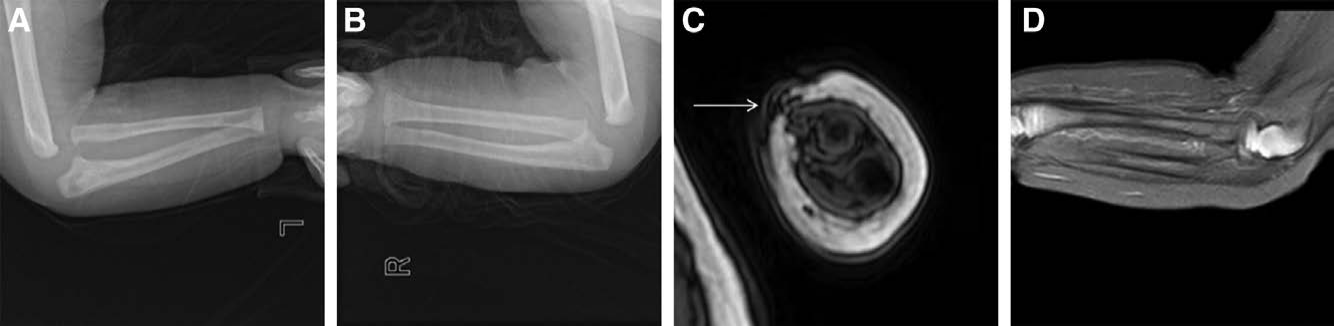
(A) The lateral X-ray of the left elbow showed anterior displacement of the radial head and a slight anterior bowing of the posterior margin of the ulna; (B) for comparison, an X-ray was performed on the right elbow, which showed normal humeroradial relationship and curve of the ulnar margin; (C) the axial T1WI inverse phase image revealed the lump as a patchy areas of low signal intensity (white arrow); (D) the sagittal PDWI-SPIR showed a slight anterior displacement of the radial head. PDWI-SPIR = Proton Density Weighted Imaging with Spectral Presaturation with Inversion Recovery.

At 3 months of age, the patient family noticed an “audible click” of the left elbow and sought medical attention again. They denied any history of trauma. Physical examination revealed normal passive elbow movements but a clicking sensation at the head of the radius during passive forearm rotation. The color and range of the original bruise faded away compared to birth, but his left wrist was still drooped naturally without any active extension movement. A momentary extension of the fingers was observed after stimulation, with overall weak strength and inability to reach neutral position in the middle finger (Fig. 2). X-rays exhibited inadequate alignment of the left humeroradial joint (Fig. 3A and B), and an MRI displayed abnormal signal intensity in the region of the lump (Fig. 3C). The humeroradial relationship seemed to be abnormal (Fig. 3D), while visualizing the radial nerve in infants on MRI proved to be challenging. We also performed electromyography on the left upper limb, which indicated damage to the left radial nerve, involving the triceps brachii. At this point, we considered the possibility that the patient may have both radial nerve palsy in the newborn and CRHD, although the latter could not be confirmed at that time. The patient received regular physical therapy including joint function training, low-frequency pulse electrical therapy, acupuncture, paraffin treatment, as well as overnight splint immobilization and regular follow-up visits.

## 3. Results

**Figure 4.**
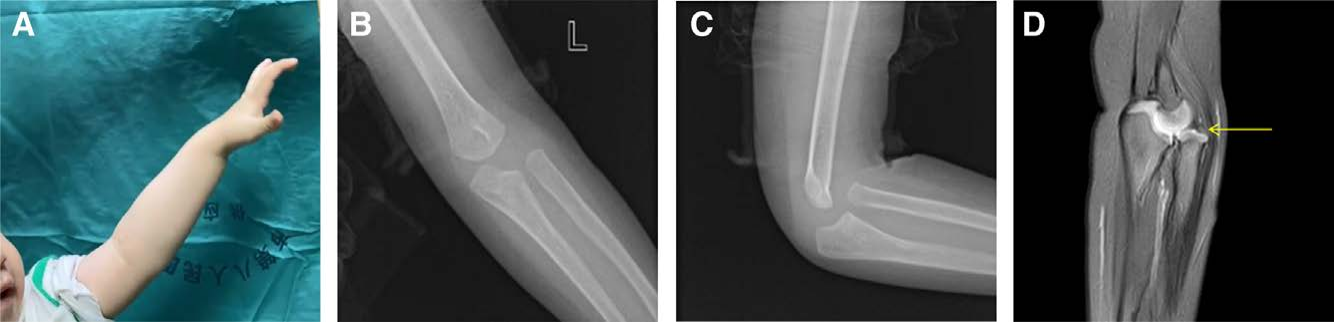
(A) The child could actively extend the wrist against gravity, approaching a neutral position, while could also extend all fingers; (B) the anteroposterior X-ray of the left elbow does not show obvious ossification center of the capitulum of the humerus, indicating possible hypoplasia; (C) the lateral X-ray of the left elbow revealed that the radial head was still displaced anteriorly; (D) the sagittal PDWI-SPIR showed that the articular surface of the radial head had appeared domed (yellow arrow), along with a subluxation of the humeroradial joint. PDWI-SPIR = Proton Density Weighted Imaging with Spectral Presaturation with Inversion Recovery.

The latest follow-up was at 16 months of age. The initial lump in the patient elbow was no longer palpable, leaving a superficial scar on the skin. The child could actively extend the wrist to a neutral position and extend all fingers (Fig. 4A). There was still a bouncing sensation in the radial head. We rechecked the X-ray and did not find obvious ossification center in the humeral head (Fig. 4B), and the radial head was still anterior dislocation (Fig. 4C). In addition, the MRI also showed a dome-shaped articular surface of the radial head, accompanied by subluxation of the humeroradialar joint (Fig. 4D), and no signal was observed from the original lump in any sequence.

## 4. Discussion

Peripheral nerve injuries most commonly affect the brachial plexus,^[1]^ which can have an impact on upper limb function in newborns. The incidence of neonatal brachial plexus injury is reported to be between 0.4 to 2.5 cases per 1000 newborns.^[2,3]^ On the other hand, cases of isolated radial nerve palsy in newborns are rare and usually detected within the first week after birth,^[4,5]^ predominantly affecting one side, with few cases being bilateral. CRHD is also rare but is the most common congenital deformity in the elbow, with an incidence ranging from 0.06% to 0.16%.^[6,7]^ Posterior dislocation is the most common (65%), followed by anterior dislocation (18%) and lateral dislocation (17%), with anterior dislocation leading to the greatest limitation of motion. The deformity is typically bilateral but can also occur unilaterally. Unlike radial nerve palsy, CRHD is not commonly seen in newborns and often remains asymptomatic until before adolescence. Early diagnosis of this deformity is challenging due to the immature ossification centers in young children, and it is usually diagnosed when there is limited elbow motion or pain.^[8]^

The patient we reported on had isolated radial nerve palsy at birth and no evidence of radial head dislocation was found. However, at 3 months of age, a suspected subluxation was observed. Considering factors such as joint laxity, muscle imbalance, and potential positional interference during X-ray imaging, a definitive diagnosis of CRHD cannot be made at this time due to the limitations of imaging examinations in providing sufficient sensitivity. It was not until 16 months of age, according to the diagnostic criteria described by McFarland,^[9]^ Mardam Bey and Ger for CRHD,^[7]^ that the dislocation was confirmed as congenital. These criteria include a dome-shaped radial head, dysplastic capitulum of the humerus, mild anterior bowing of the ulnar posterior margin, no history of trauma, and the presence of other congenital anomalies. Although it is referred to as dislocation, the radial head is actually in a partially dislocated state at this time. It is uncertain how long this state will last or whether it will eventually progress to a complete dislocation. Some researchers have described cases where a subluxation progressed into a complete dislocation or remained in a partially dislocated state.^[10]^

The most challenging and crucial differential diagnosis for radial nerve palsy in the newborn is brachial plexus birth palsy. Due to misdiagnosis in some cases, the incidence of radial nerve palsy in the newborn may be underestimated. Brachial plexus injury can result in weakened grip strength or limited shoulder and elbow movement, while radial nerve injury can cause weakness in wrist and finger extensors. However, shoulder and elbow movement, as well as finger flexion, remain normal in cases of radial nerve injury, which is the main distinguishing factor between the 2 diseases. Another diagnostic indicator of radial nerve palsy in the newborn is characteristic skin manifestations, such as nodules, bruise, and skin dimpling in the lateral region of the humerus or near the lateral epicondyle, which is the most vulnerable area for radial nerve compression. The patient we reported had evident bruise and palpable lump on the anterolateral side of the left elbow at birth, and an abnormal signal in the lump was confirmed by MRI at 3 months of age. Although we couldn’t clearly distinguish the radial nerve from the images, the area below the lump corresponds to the course of the radial nerve on the surface of the radial head, indicating a probable compression. Morgan first associated this type of lump with radial nerve palsy in the newborn in 1948.^[11]^ In a study of the largest series of 25 cases by Alsubhi et al, 17 cases (68%) had subcutaneous nodules.^[12]^ Biopsy confirmed fat necrosis in the nodules,^[13,14]^ suggesting that isolated compression was the underlying cause of radial nerve palsy in the newborn, which may be related to abnormal intrauterine position or delivery process, as many cases involved prolonged labor or difficult deliveries.^[15,16]^ The exact time of onset of radial nerve injury cannot be determined, but it may occur in the late stages of pregnancy, during delivery, or both.^[1]^ Electrophysiological findings have revealed the occurrence of acute denervation within the first week after birth, which is typically observed at least 10 days after nerve injury.^[12]^ This suggests that the injury may occur prior to delivery,^[4,5]^ leading some scholars to refer to this condition as “Congenital Radial Nerve Palsy.” The mother of our case underwent a cesarean section, but the baby still developed radial nerve palsy, which also proves that the injury occurred before delivery. The electromyography at the age of 3 months showed that the left radial nerve damage involved the triceps brachii, and this phenomenon has also been reported in the literature, suggesting that it may be due to the involvement of the triceps brachii branch caused by radial nerve palsy.^[5,15–17]^

The pathogenesis of CRHD is not well understood. Some patients have a clear family history of the condition, but there is no definite inheritance pattern. Approximately 60% of cases of CRHD are related to other congenital upper limb deformities,^[7,18]^ which is consistent with the case we reported. The patient underwent genetic testing at 10 months of age, which revealed a possible diagnosis of Klinefelter syndrome. Almquist et al first reported a case of Klinefelter syndrome with radial head dislocation in 1969,^[19]^ and multiple authors have reported that this syndrome is often associated with some skeletal abnormalities. Recent studies provide convincing evidence that altered HOX D activity/expression is a primary factor of CRHD.^[20]^

The prognosis for radial nerve palsy in the newborn and CRHD reported in the literature is generally good. Radial nerve palsy in the newborn typically only requires joint mobilization training and physical therapy, along with nighttime wrist splinting to prevent flexion contractures. There is clear evidence that even without any intervention, this condition can fully recover without any residual effects.^[4,16]^ So far, almost all cases of radial nerve palsy in the newborn in the literature have shown spontaneous recovery. In a review conducted by Böhringer Elisabeth and Weber Peter on 55 cases, 52 (94%) fully recovered within 4 days to 2 years.^[21]^ In the series reported by Alsubhi et al, all patients recovered completely within 1 week to 6 months, with 72% of children fully recovering within 2 months.^[12]^ Only 1 child reported by Richardson underwent surgical treatment without improvement at 18 months of observation.^[22]^ The last follow-up of the case we reported was 16 months of age and only received a wrist splint and physical therapy. The child still demonstrates habitual wrist and finger drooping at rest, indicating that complete recovery has not been achieved yet. However, with active movement or an external stimulation, he is able to extend the wrist to a neutral position, and fingers and thumb extension are normal. Currently, the recovery time for our case seems longer than the average reported in the literature, but the child is still gradually improving, so we believe that there is no indication for surgical intervention at this time, and continued physical therapy and follow-up are appropriate. There are also cases in the literature with slower recovery rates. For example, in a summary of 4 cases by Monica et al, 1 case had weak extension strength of the middle finger and only recovered at the age of 2, while another still had skin depressions on the lateral aspect of the distal humerus at the age of 6.^[16]^

For CRHD, authors generally recommend conservative treatment and observation, as even without any treatment, cases with significant symptoms in adulthood are rare.^[23–25]^ Even if diagnosed in newborns, there are no treatment indications. If pain or limited movement occurs later on, radial head excision surgery can be performed after the age of 15.

The limitations of this study include the relatively short follow-up period, which limits our understanding of long-term outcomes and potential complications. Additionally, as a case report, the findings should not be considered as universally applicable guidelines, but rather as experiential reference.

## 5. Conclusions

If a neonate exhibits limited extension in the wrist and fingers, but normal flexion, along with normal shoulder and elbow movement, and is accompanied by characteristic skin lesions around the elbow, there should be a high suspicion of radial nerve palsy in the newborn. Early initiation of physical therapy can be beneficial in restoring nerve function, and the vast majority of cases can achieve complete recovery without any sequelae. Conversely, the diagnosis of CRHD is often difficult during the neonatal period, but regardless of the timing of diagnosis, the vast majority of cases only require observation.

## Author contributions

**Conceptualization:** Yunlong Li.

**Data curation:** Yunlong Li, Yongyao Jiang, Weiyue Zhu.

**Formal analysis:** Guoxin Nan, Jiahui Chen.

**Investigation:** Yunlong Li, Yongyao Jiang.

**Methodology:** Guoxin Nan, Jiahui Chen.

**Resources:** Yunlong Li.

**Supervision:** Jiahui Chen.

**Writing – original draft:** Yunlong Li.

**Writing – review & editing:** Yunlong Li.
